# Immunotherapies for advanced hepatocellular carcinoma

**DOI:** 10.3389/fphar.2023.1138493

**Published:** 2023-03-21

**Authors:** Li-Yang Sun, Kang-Jun Zhang, Ya-Ming Xie, Jun-Wei Liu, Zun-Qiang Xiao

**Affiliations:** ^1^ The Second School of Clinical Medicine, Zhejiang Chinese Medical University, Hangzhou, China; ^2^ Cancer Center, General Surgery, Department of Hepatobiliary and Pancreatic Surgery and Minimally Invasive Surgery, Zhejiang Provincial People’s Hospital, Affiliated People’s Hospital, Hangzhou Medical College, Hangzhou, Zhejiang, China

**Keywords:** hepatocellular carcinoma, immunotherapy, PD-1, PD-L1, CTLA-4

## Abstract

Primary liver cancer is the second leading cause of tumor-related deaths in China, with hepatocellular carcinoma (HCC) accounting for 80%–90% of these. Since there is a lack of symptoms in the early stages of HCC, a large proportion of patients were identified with unresectable HCC when diagnosed. Due to the severe resistance to chemotherapy, patients with advanced HCC were traditionally treated with systematic therapy in the past decades, and the tyrosine kinase inhibitor (TKI) sorafenib has remained the only treatment option for advanced HCC since 2008. Immunotherapies, particularly immune checkpoint inhibitors (ICIs), have shown a strong anti-tumor effect and have been supported by several guidelines recently. ICIs, for example programmed cell death-1 (PD-1) inhibitors such as nivolumab and pembrolizumab, programmed cell death ligand 1 (PD-L1) inhibitors such as atezolizumab, and cytotoxic T-lymphocyte-associated protein 4 (CTLA-4) inhibitors such as ipilimumab, the ICI-based combination with TKIs, and VEGF-neutralizing antibody or systematic or local anti-tumor therapies, are being further studied in clinical trials. However, immune-related adverse events (irAEs) including cutaneous toxicity, gastrointestinal toxicity, and hepatotoxicity may lead to the termination of ICI treatment or even threaten patients’ lives. This review aims to summarize currently available immunotherapies and introduce the irAEs and their managements in order to provide references for clinical application and further research.

## Introduction

Liver cancer is a concerning health challenge and is the sixth most common malignancy and the fourth leading cause of cancer-related mortality worldwide (Villanueva, 2019; Llovet et al., 2021a). HCC, which is generally attributed to the background of chronic liver diseases including hepatitis B virus (HBV) or hepatitis C virus (HCV) infection and alcoholic liver disease or non-alcoholic fatty liver disease (NAFLD), accounts for over 90% of liver cancers (Younossi et al., 2018; Llovet et al., 2021b). Although the incidence rates of HCC have decreased due to the coverage of HBV vaccines and anti-viral therapies in some regions, the global incidence of HCC continues to rise, resulting in at least 1,000,000 HCC cases annually by 2025 (Llovet et al., 2021a; Sung et al., 2021). Unfortunately, due to the lack of symptoms and physical characteristics of HCC patients, as well as the unsatisfactory HCC surveillance accuracy and popularity, potentially curative treatment is not possible for over 80% of patients at the time of diagnosis (Zongyi and Xiaowu, 2020). Due to the severe and broad resistance to cytotoxic chemotherapy, systemic therapy was a controversial option for patients with advanced HCC before 2008. After years of waiting and many unsuccessful clinical trials, Llovet et al. (2008) demonstrated the anti-tumor effect of sorafenib as an oral multi-kinase inhibitor in a phase III trial, the SHARP study. Sorafenib was the first systemic therapy for HCC, prolonging survival by a few months. Although the survival benefit of using sorafenib is not clinically meaningful, the viable option for advanced HCC was limited to sorafenib alone for 10 years until the emergence of lenvatinib, which not only showed an overall survival (OS) that was not inferior to sorafenib but also improved all secondary endpoints (Al-Salama et al., 2019). Moreover, regorafenib was also approved as the second-line therapeutic setting for advanced HCC (Llovet et al., 2018).

In addition to TKIs including sorafenib, lenvatinib, and regorafenib, immunotherapy is gaining continued traction in treating advanced HCC (Fulgenzi et al., 2021). Based on the cancer immunosurveillance hypothesis postulating that evasion from immune control is an essential feature of cancer, immune checkpoint molecules including PD-1, PD-L1, and CTLA-4 were further studied (Brahmer et al., 2010; 2012; Pardoll, 2012; Topalian et al., 2012; Zitvogel et al., 2016a; Zitvogel et al., 2016b). In fact, ICIs have been proven to be an efficacious anti-cancer strategy in other solid cancers, e.g., non-small-cell lung cancer (NSCLC), renal cancer, and melanoma (Larkin et al., 2015; Amin and Hammers, 2018; Mazieres et al., 2019). Recent clinical trials have also discovered the prolonged survival of HCC patients using ICIs, showing the promising curative effect of ICIs toward HCC (Finn et al., 2020a; Greten et al., 2021; Yau et al., 2022). As a breakthrough, the combination of atezolizumab plus bevacizumab was introduced into the first-line therapies for advanced HCC, which has provided patients with a hopeful option (Llovet et al., 2021b).

## Mechanisms of immunotherapy

**FIGURE 1:**
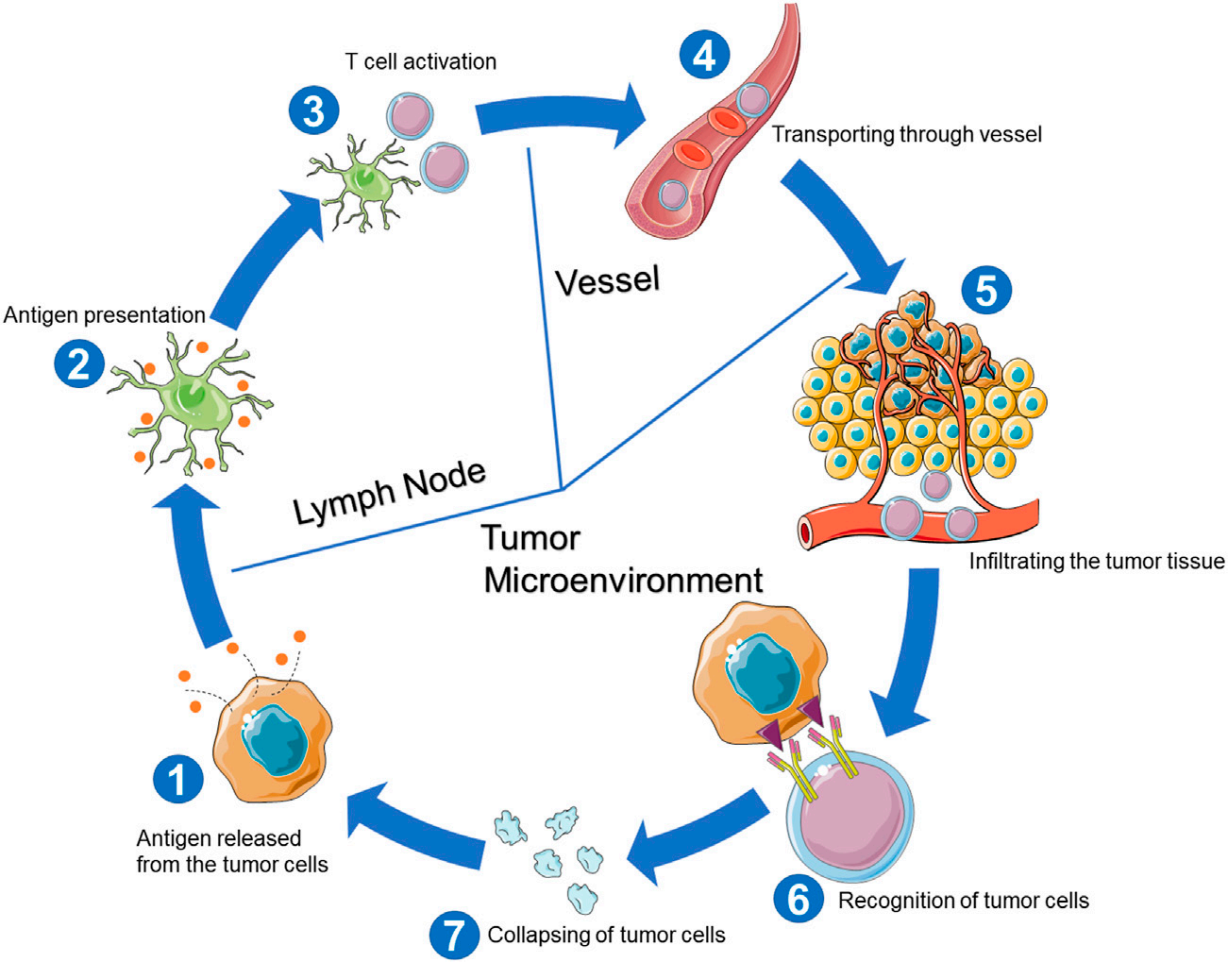
Cancer-immunity cycle for killing tumor cells.

In the normal cancer-immunity cycle for killing tumor cells (Figure 1), the antigens from tumor cells are first captured and further processed by dendritic cells (DCs). Second, captured antigens are presented to T cells to activate the T-cell responses against the cancer-specific antigens (Chen and Mellman, 2013). After assembling in the tumor tissue and infiltrating the tumor bed, T cells specifically recognize and bind tumor cells and then kill the targeted tumor cells (Chen and Mellman, 2013). However, in tumor patients, the cancer-immunity cycles fail to run optimally, leading to tumor development and even endangering the host’s life.

**FIGURE 2:**
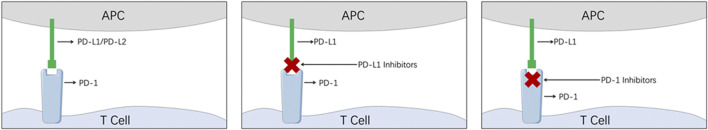
Illustration of the mechanism of PD-1/PD-L1 inhibitors. (APC, antigen-presenting cell; PD-1, programmed cell death-1; PD-L1, programmed cell death ligand 1).

Belonging to the immunoglobulin super family, PD-1 is a transmembrane coinhibitory receptor primarily expressed on the surface of activated T cells and NK cells (Huang et al., 2021) as the ligands to PD-1, PD-L1 (B7-H1 or CD274), and PD-L2 (B7-DC or CD273) are expressed on the surface of tumor cells (Figure 2) (Huang et al., 2021). Once the tumor cells are detected by the T cells, the overexpressed PD-L1/2 from the tumor cells engages with PD-1 on the T cells, and the physiological inhibitory pathways will therefore be hijacked by the tumor cells to escape the host immune surveillance system (Huang et al., 2021). After numerous attempts, the PD-1/PD-L1 inhibitors were proved to have the ability to remove the coinhibitory signal by blocking PD-1 or PD-L1, and rebuild the normal immune system surveillance kill tumor cells (Sharma and Allison, 2015).

**FIGURE 3:**
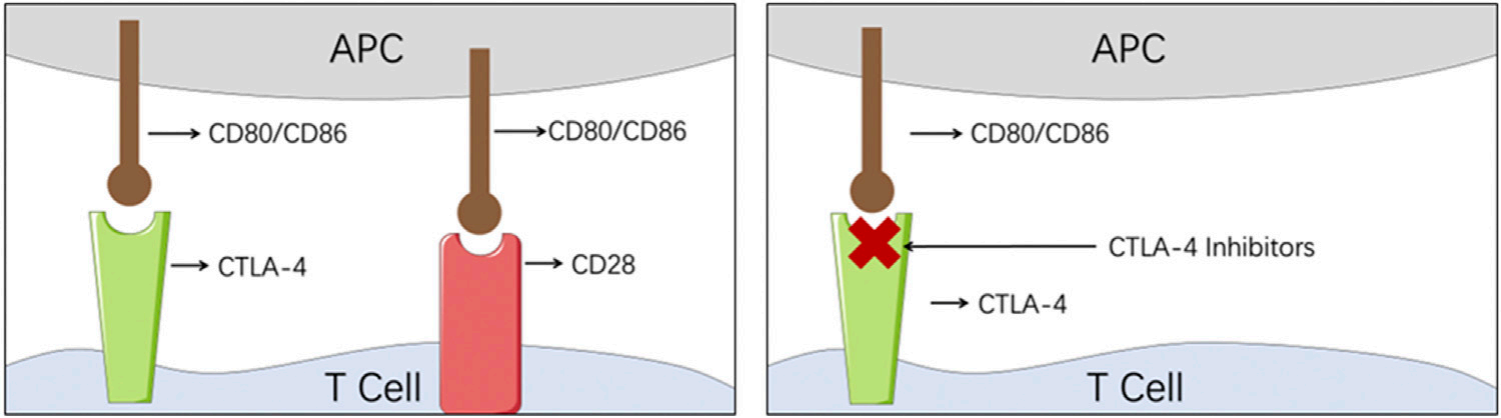
Illustration of the mechanism of CTLA-4 inhibitors. (APC, antigen-presenting cell; CTLA-4, cytotoxic T-lymphocyte-associated protein 4).

CTLA-4, as a member of the CD28 immunoglobulin subfamily, is also mainly expressed on the T cells (Figure 3). When CTLA-4 engages with its ligands, CD80 and CD86, similarly to CD28, the coinhibitory response will be activated and the tumor cell will escape the host immune surveillance system (Rowshanravan et al., 2018; Xu et al., 2018). On the contrary, when CD80 and CD86 engage with CD28, the costimulatory response is initiated (Rowshanravan et al., 2018; Xu et al., 2018). Therefore, by blocking the checkpoint CTL4-4, CTLA-4 inhibitors managed to repair the collapsed immune surveillance system.

## PD-1/PD-L1 inhibitors

The engagement of PD-1 expressed on the surface of activated CD8^+^ T cells with PD-L1 expressed by HCC cells not only averts the excessive activation of T cells, decreasing tumor-killing efficiency by transmitting inhibitory signals, but also weakens proliferation and cytolytic activity, followed by the defects or even deletion of cytokine production, eventually leading to an exhausted T-cell phenotype (Wherry, 2011). With regard to the mechanisms of the PD-1 engagement with PD-L1 in the development of HCC, PD-1 and PD-L1 inhibitors are widely recognized as the backbone of systemic therapies for HCC, and several main randomized clinical trials are shown in Table 1.

**TABLE 1:** Main randomized clinical trials of ICIs for advanced HCC.

Name	Treatment	Study phase	Control group	Primary endpoint	ORR, %	Median OS, months	Median PFS, months
CheckMate 040	Nivolumab	I/II	None	Safety and ORR	20	15.6	4.0
CheckMate 040	Nivolumab plus ipilimumab	I/II	None	Safety, tolerability, and ORR	32	22.8	NR
KEYNOTE-224	Pembrolizumab	II	None	ORR	17	12.9	4.9
KEYNOTE-240	Pembrolizumab	III	Placebo	OS and PFS	18.3 vs. 14.4, *p* < 0.001	13.8 vs. 10.6, *p* = 0.024	3.0 vs. 2.8, *p* = 0.002
CheckMate 459	Nivolumab	III	Sorafenib	OS	15 vs. 7, *p* = NR	16.4 vs. 14.8, *p* = 0.052	3.7 vs. 3.8, *p* = NS
IMbrave150	Atezolizumab plus bevacizumab	III	Sorafenib	OS and PFS	30 vs. 11, *p* < 0.001	19.2 vs. 13.4, *p* < 0.001	6.8 vs. 4.3, *p* < 0.001
COSMIC-312	Cabozantinib plus atezolizumab	III	Sorafenib	OS and PFS	13 vs. 6, *p* = NR	15.4 vs. 15.5, *p* = 0.44	6.8 vs. 4.2, *p* = 0.001

ICI, immune checkpoint inhibitor; NR, not reported; NS, not significant; ORR, objective response rate; OS, overall survival; PFS, progression-free survival.

In 2007, according to the results of the CheckMate 040 trial, nivolumab was granted accelerated approval by the US Food and Drug Administration (FDA) as a PD-1 inhibitor for treating advanced HCC after the failure of sorafenib (Chiew Woon et al., 2020). In the CheckMate 040 trial, 214 patients in the dose-expansion phase and 48 patients in the dose-escalation phase were enrolled (El-Khoueiry et al., 2017). According to the Response Evaluation Criteria in Solid Tumors (RECIST) 1.1 criteria, an objective response rate (ORR) of 20% (95% CI: 15%–26%) was shown in the dose-expansion phase at the nivolumab dose of 3 mg/kg every 2 weeks and an ORR of 15% (95% CI: 6%–28%) was shown in the dose-escalation phase (El-Khoueiry et al., 2017). Among 48 patients in the dose-escalation phase, the median duration of response to nivolumab was 17 months (95% CI: 6–24 months), and among responders, a 2-year survival rate of over 80% was observed (El-Khoueiry et al., 2017).

The efficacy of nivolumab was further evaluated in the CheckMate 459 trial by comparing it with sorafenib, which was the first systemic agent approved for the treatment of HCC (Man et al., 2021; Yau et al., 2022). In this randomized, open-label, phase III clinical trial, 743 patients across 22 countries and regions were finally selected and randomly assigned into two cohorts (nivolumab, n = 371; sorafenib, n = 372) (Yau et al., 2022). At the follow-up after 22.8 months, the nivolumab cohort achieved a median OS of 16.4 months (95% CI: 13.9–18.4 months) *versus* the sorafenib cohort that achieved a median OS of 14.7 months (95% CI: 11.9–17.2 months). Despite an extra 2 months of OS time, with a *p*-value of 0.075, the CheckMate 459 trial did not meet the primary boundary. However, given the fact that at least 31% of patients from the sorafenib cohort had received ICIs after sorafenib treatment, as well as the secondary endpoints favoring nivolumab over sorafenib, the study still concluded that nivolumab was superior to sorafenib, with encouraging long-term survival, durable clinical activity of response frequency and durability, less immune-related adverse events, and clinically meaningful improvements in health-related quality of life (Sangro et al., 2021; Yau et al., 2022).

Similar to nivolumab, pembrolizumab is another fully humanized PD-1 monoclonal antibody inhibitor. A year after the approval of nivolumab, considering the results from a non-randomized, multicenter, open-label, phase II trial “KEYNOTE-224” reported in 2018, the FDA approved pembrolizumab for the treatment of advanced HCC after sorafenib failure or intolerance (Zhu et al., 2018). After recruitment and screening, 104 patients with advanced HCC after sorafenib treatment were finally enrolled into this study. The primary endpoint was an objective response, and it was shown that the objective response was observed in 18 patients (17%), among which the best overall responses were complete response from one patient (1%) and partial responses from 17 patients (16%) (Zhu et al., 2018). Meanwhile, the median OS was 12.9 months (95% CI: 11.9–17.2 months), the median progression-free survival (PFS) was 4.9 months (95% CI: 3.9–8.0 months), the 1-year PFS rate was 28% (95% CI: 19%–37%), and the 1-year OS rate was 54% (95% CI: 44%–63%) (Zhu et al., 2018).

However, the trial “KEYNOTE-224” was a non-randomized study without a control group, and the results were further validated in a large randomized, phase III trial “KEYNOTE-240” (Finn et al., 2020b). In 2020, the results of KEYNOTE-240 were reported. The efficacy of pembrolizumab was further evaluated by comparing with the control cohort using best supportive care (BSC) or placebo plus BSC, and the primary endpoint was OS and PFS. A total of 413 advanced HCC patients from 119 institutions across 27 countries were finally recruited and divided into the pembrolizumab cohort (n = 278) and the placebo cohort (n = 135) (Finn et al., 2020c). It was reported that the median OS of the pembrolizumab cohort was 13.9 months (95% CI: 11.6–16.0 months), which was better than the median OS of 10.6 months (95% CI: 8.3–13.5 months) from the placebo cohort with a *p*-value of 0.0238. In terms of tumor progression, the pembrolizumab cohort showed a median PFS of 3.0 months (95% CI: 2.8–4.1 months), which was superior to that of 2.8 months (95% CI: 1.6–3.0 months) from the placebo cohort (*p* = 0. 0022) (Finn et al., 2020a). Although both the OS and PFS were improved after pembrolizumab treatment compared to the placebo cohort, the trial “KEYNOTE-240” was still judged as a failure as it did not meet the prespecified statistical endpoints.

As shown previously, the ORR of several PD-1 inhibitors was only 15%–20%, and the first-line monotherapy trial “CheckMate 459” and the second-line monotherapy trial “KEYNOTE-240” were both declared failures (Finn et al., 2020b; Yau et al., 2022). It was not until the emergence of IMbrave150 that hope was revived for the systematic treatment of HCC. With the publication of this global, open-label, phase III randomized trial, the combination of the PD-L1 inhibitor, atezolizumab, and the anti-VEGF monoclonal antibody, bevacizumab, was highly expected as a novel strategy for unresectable HCC treatment (Finn et al., 2020c).

Before IMbrave 250, atezolizumab treatment had been validated as a superior option to platinum-based chemotherapy for NSCLC patients with high PD-L1 expression (Herbst et al., 2020). In this trial, a total of 501 patients with locally advanced metastatic or unresectable HCC were finally enrolled, of which 336 (67.1%) patients were randomly assigned to receive atezolizumab plus bevacizumab, while 165 (32.9%) patients were included in the sorafenib cohort. Defining OS and PFS as the primary endpoints, patients of the atezolizumab–bevacizumab cohort conducted better estimated survival rates at timepoints of 6 months (84.8% *versus.* 72.2%) and 12 months (67.2% *versus.* 54.6%) compared to the sorafenib cohort (Finn et al., 2020a). Meanwhile, the atezolizumab–bevacizumab cohort also had a significantly longer PFS than the sorafenib cohort (median, 6.8 months *versus.* 4.3 months, *p* < 0.001). Furthermore, the PFS at 6 months in the atezolizumab–bevacizumab cohort was 54.5%, which was much higher than 37.2% in the sorafenib cohort. Not only the primary endpoints but also the secondary endpoints of the atezolizumab–bevacizumab cohort performed better than the sorafenib cohort. The confirmed ORR was 27.5% (95% CI: 22.5%–32.5%) in the atezolizumab–bevacizumab cohort, which was significantly superior to that of 11.9% (95% CI: 7.4%–18.0%) in patients treated with sorafenib (*p* < 0.001).

## CTLA-4 inhibitors

Similar to PD-1, CTLA-4 is another member of the immunoglobulin-related receptor family regulating various aspects of T-cell immune functions (Zhang et al., 2019). CTLA-4 is mainly expressed in regulatory T cells, which transmits a negative signal directly in effector T cells and regulates the negative immune responses of T cells (Lisi et al., 2022). Therefore, CTLA-4 has been envisioned as a target of monoclonal antibodies for cancer immunotherapy and CTLA-4 inhibitors. To enhance its anti-tumor effect, nowadays, the CTLA-4 inhibitors are widely used in combination with other ICIs (Yau et al., 2020; Pinato et al., 2021a).

Ipilimumab was the first CTLA-4 inhibitor approved in 2010 for metastatic melanoma (Hodi et al., 2010). Meanwhile, tremelimumab was the first CTLA-4 inhibitor used for HCC treatment (Sangro et al., 2013). In the clinical trial conducted by Sangro et al., 20 HCV-positive patients with inoperable HCC were enrolled and received intravenous tremelimumab at a dose of 15 mg/kg on day 1 of every 90-day cycle until tumor progression and occurrence of unacceptable toxicities. The trial showed that under tremelimumab treatment, patients with inoperable HCC achieved a median OS of 8.2 months and a median time-to-progression (TTP) of 6.48 months. Moreover, the 6-month survival rate was 64% and the 1-year survival rate was 43%.

## Exploration of ICI combinations

Apart from the combination of atezolizumab and bevacizumab, several studies investigated the possibility of combining ICIs of different targets. As it was mentioned previously, CTLA-4 ICIs were usually used in combination with PD-1/PD-L1 ICIs. In the CheckMate 040 trial, a total of 148 patients were enrolled to receive the combination of ipilimumab and nivolumab (Yau et al., 2020). In this multicenter, open-label, phase I/II study, patients were randomly divided into three arms (50 in arm A and 49 each in arms B and C). The dose of ipilimumab–nivolumab differed across different arms. Patients in arm A were treated with nivolumab 1 mg/kg plus ipilimumab 3 mg/kg every 3 weeks, followed by nivolumab 240 mg every 2 weeks; patients in arm B were treated with nivolumab 3 mg/kg plus ipilimumab 1 mg/kg every 3 weeks, followed by nivolumab 240 mg every 2 weeks; and patients in arm C were treated with nivolumab 3 mg/kg every 2 weeks plus ipilimumab 1 mg/kg every 6 weeks. After follow-ups, arm A showed the highest ORR of 32% (95% CI: 20%–47%) compared with 27% (95% CI: 15%–41%) in arm B and 29% (95% CI: 17%–43%) in arm C and the longest median OS of 22.8 months *versus* 12.5 months and 12.7 months in arms B and C, respectively.

In addition to the combination of different ICIs, the combination of ICIs and TKIs is also a potentially effective treatment for advanced HCC. TKIs play an anti-tumor role by blocking several angiogenic pathways and further maintaining the consequent stability of the vascular endothelium in the tumor bed (Wong et al., 2015). TKIs, along with ICIs, have been considered the cornerstone for systematic HCC treatment. Since 2007, several TKIs, including sorafenib and lenvatinib, have been approved for the systemic treatment of advanced HCC (Al-Salama et al., 2019). Research on exploring the efficacy of the combination of TKIs and ICIs in the treatment of advanced HCC has never stopped. An international, open-label, randomized clinical phase III trial named COSMIC-312, which studied the combination of cabozantinib and atezolizumab, was recently published (Antonella et al., 2022; Kelley et al., 2022). A total of 837 advanced HCC patients have been enrolled and randomly treated with cabozantinib–atezolizumab, sorafenib alone, or cabozantinib alone in a 2:1:1 ratio. Researchers assessed the PFS in accordance with RECIST 1.1 that was assessed by a blinded and independent committee for the first 372 patients from the cabozantinib–atezolizumab cohort or sorafenib cohort and OS in all the patients from the cabozantinib–atezolizumab cohort or sorafenib cohort as the dual primary endpoints of this study. It was reported that the combination treatment cohort achieved a median PFS of 6.8 months (95% CI: 5.6–8.3 months) *versus* 4.2 months (95% CI: 2.8–7.0 months) in the sorafenib cohort with a statistically significant *p*-value of 0.0012. However, in the interim analysis, the median OS in the cabozantinib–atezolizumab cohort was 15.4 months (95% CI: 13.7–17.7 months), while the median OS in the sorafenib cohort was 15.5 months (12.1- not estimable) with a *p*-value of 0.44. Additionally, in subgroups with more advanced HCC, an improved PFS was also observed, and further studies to evaluate the efficacy of cabozantinib plus atezolizumab are still needed.

## Ongoing clinical trials

Needless to say, the research focusing on the immune checkpoint inhibitors is far from over, and there are abundant clinical trials ongoing, exploring efficient immunotherapies. An abstract of the clinical trial “RATIONALE 301” exploring the efficiency of tislelizumab *versus* sorafenib for advanced HCC was reported recently. The study revealed that patients receiving tislelizumab showed an OS not inferior to that of those receiving sorafenib (15.9 months *versus.* 14.1 months), and the tislelizumab cohort showed a better ORR (14.3% *versus.* 5.4%). Meanwhile, fewer patients in the tislelizumab cohort experienced irAEs, and fewer patients suffered irAEs that led to discontinuation or dosing adjustment. Another clinical trial (NCT03764293) evaluated the efficiency and safety of the combination of camrelizumab with rivoceranib for unresectable HCC compared with those of sorafenib. The combination cohort showed both significantly longer median OS (22.1 months *versus.* 15.2 months) and median PFS (5.6 months *versus.* 3.7 months). Notably, the combination of camrelizumab with rivoceranib achieved the longest median OS among all the phase III clinical trials for advanced HCC, and this combination has the potential to be another first-line treatment option.

## Adverse events after ICI treatments

Although the therapeutics for advanced HCC were reshaped, the immune-modulatory therapy inevitably leads to immune system imbalance and a series of irAEs including cutaneous toxicity, gastrointestinal toxicity, hepatotoxicity, and thyroiditis (Mitchell et al., 2013; Khoja et al., 2017; Wu et al., 2017; Barroso-Sousa et al., 2018; Kurimoto et al., 2020; Pinato et al., 2021b). Unfortunately, the precise mechanism of irAEs still remains unclear. These irAEs tend to appear after 8 weeks of ICI treatment, and most of them are typically reversible and controllable, but occasionally they lead to withdrawal or fatal outcomes. Therefore, monitoring and managing such irAEs are also an essential part of ICI therapeutic strategies, and most clinical trials considered the occurrence of irAEs as one of the endpoints of the whole trials.

In terms of irAEs in all the cancers, irAEs after PD-1 or PD-L1 inhibitor treatment are dose-independent, while in those treated with the CTLA-4 inhibitor, the occurrence of irAEs tends to be dose-dependent (Bertrand et al., 2015; Wang et al., 2019). Two independent meta-analyses on PD-L1 and CTLA-4 reached similar conclusions that the most common target organs for irAEs are skin, followed by the gastrointestinal tract and liver (Bertrand et al., 2015; Wang et al., 2018). Meanwhile, for patients treated with PD-1/PD-L1 inhibitors in combination with chemotherapy, radiotherapy, immunotherapy, or targeted therapy, a meta-analysis identified that the most common irAEs of all grades were anemia (45.4%), fatigue (combination with targeted therapy) (34.3%), fatigue (combination with targeted therapy) (26.4%), and dysphagia ((30.0%), respectively, and the most common irAEs of grade 3 or higher were neutropenia (19.6%), hypertension (9.3%), a high level of lipase (7.2%), and lymphopenia (10.3%) (Zhou et al., 2021). However, due to the unique liver immunobiology and underlying liver diseases such as cirrhosis and viral hepatitis in HCC patients, the symptoms of irAEs were always covered or ignored, which poses a major challenge to the safe use of ICIs for advanced HCC patients.

Cutaneous toxicity is the most common and obvious irAEs after ICI treatment. Generally, cutaneous toxicity mostly manifesting as rash and pruritus occurs within 2 weeks after the first dose. Fortunately, less than 1% of patients receiving monotherapies and 4% of patients receiving combination therapies develop skin irAEs of grade 3 or higher (Sangro et al., 2020). According to previous studies, rash occurred in 15%–30% of patients receiving nivolumab alone, 8%–10% of patients receiving pembrolizumab alone, and 17%–29% of patients receiving a combination of nivolumab and ipilimumab. Meanwhile, pruritus occurred in 20%–27% of patients in the nivolumab cohort, 12%–18% of patients in the pembrolizumab cohort, and 30%–45% of those treated with the combination. For patients with dermatological problems after receiving ICIs, first of all, pre-existing skin conditions, chronic liver disease-related skin disorder, or any other causes of skin disorder should be identified and ruled out. For patients with cutaneous involvement of grade 1 or 2, ICI treatment can be continued after administering triamcinolone 0.1% along with antihistamine treatment. For patients with more severe symptoms (grade 3), systemic hormone therapies, such as oral prednisolone, should be given at a dose of 1–2 mg/kg on the basis of the aforementioned topical therapy. For patients with grade 4 or life-threatening skin disorders, ICI treatment should be terminated immediately and methylprednisolone should be given at a dose of 1–2 mg/kg (Brahmer et al., 2018).

Gastrointestinal toxicity in patients after ICI treatments usually manifests as diarrhea and colitis (Vogl et al., 2011; Nielsen et al., 2022). Generally, diarrhea and colitis are commonly diagnosed at 6–8 weeks, following the initiation of ICIs. For overall cancer populations, a recently published meta-analysis showed that the incidence rates of diarrhea of grade 1–4 and grade 3–4 after administering pembrolizumab at a dose of 200 mg every 3 weeks were 9.5% and 0.3%, and the incidence rates of colitis of grade 1–4 and grade 3–4 were 1.3% and 0.4%, respectively (Nielsen et al., 2022). Meanwhile, at the standard flat dose of nivolumab of 240 mg every 2 weeks, the incidence rates of diarrhea of grade 1–4 and grade 3–4 were 11.6% and 0.04%, and the incidence rates of colitis of grade 1–4 and grade 3–4 were 0.2% and 0.0%, respectively (Nielsen et al., 2022). For patients receiving a 1,200 mg dosage of atezolizumab every 3 weeks, the incidence rate of grade 1–4 and grade 3–4 diarrhea was 8.8% and 0.1%, and 0.6% and 0.3% for grade 1–4 and grade 3–4 colitis, respectively. For advanced HCC patients after ICI treatments, the incidence of diarrhea and colitis is consistent with that of the overall tumor populations. Similar to managing cutaneous toxicity, the first step in dealing with ICI-related gastrointestinal toxicity is identifying the cause of diarrhea and colitis including underlying diseases or medications that induce diarrhea, such as lactulose. Generally, colonoscopy still remains the gold diagnostic standard of gastrointestinal toxicity and contributes to severity assessment grading. As for the treatments, once gastrointestinal toxicity is identified, severity grading should be assessed by symptoms or colonoscopy first. For diarrhea of grade 1, no special treatment is needed except strengthening monitoring, and ICI treatment can be continued. Symptomatic treatments, such as parenteral administration of fluids and electrolytes, are warranted. Oral corticosteroids at a dose of 0.5–1 mg/kg should be given if diarrhea or colitis of grade 2 persisted for over 3 days, and intravenous corticosteroids are needed for gastrointestinal toxicity of grade 3 or higher. Meanwhile, ICI treatments should be terminated when patients are diagnosed with diarrhea or colitis of grade 2–3, and ICI treatment should be terminated permanently when gastrointestinal toxicity of grade 4 is identified.

Since patients with advanced HCC are usually diagnosed with underlying chronic liver diseases or liver dysfunction, hepatotoxicity, which always manifests as hepatitis or elevations of liver enzymes after ICI treatments, is a relatively frequent irAE. Compared with other types of tumors, including melanoma and NSCLC, a higher proportion of liver enzyme increase occurred after ICI treatment in HCC (Vogl et al., 2011; Brown et al., 2017; Lleo et al., 2019; De Martin et al., 2020). Elevations of liver enzymes were found in 13% of patients receiving pembrolizumab in the trial “KEYNOTE-224” and 16% of patients receiving nivolumab plus ipilimumab in the trial “CheckMate 040” (Zhu et al., 2018; Yau et al., 2020). Patients after ICI treatments should undergo regular liver function examinations as the hepatitis or liver dysfunction tend to be asymptomatic and progress rapidly to liver failure at later stages. Hepatotoxicity commonly occurs at 4–12 weeks after the initial ICI treatment. For patients with ICI-related hepatotoxicity, steroid therapy is not necessarily required, and ICIs can be continued or delayed if patients were identified with asymptomatic liver enzyme elevation or irAEs of grade 1–2. As for patients with hepatotoxicity of grade ≥3, the level of liver enzymes mostly returns to normal after timely steroid therapy, and ICI can be reintroduced when the level of aminotransferases declines or returns to baseline levels (De Martin et al., 2018).

Thyroiditis related to ICI treatment is generally assumed as the main etiology of thyroid dysfunction, which is the most commonly observed endocrine gland irAE (Muir et al., 2021). The symptoms of thyroid dysfunction vary, including hyperthyroidism and hypothyroidism, of which hypothyroidism accounts for the majority. The diagnosis of thyroid dysfunction is mainly based on the comparison of thyroid hormone levels before and after ICI treatment (Illouz et al., 2018). The incidence of thyroid dysfunction varies due to the different types of ICIs. It was reported in a phase III clinical trial that the incidence of hypothyroidism was 13.0% in advanced HCC patients after receiving a PD-1 inhibitor and 22.2% after receiving a combination of PD-1 inhibitor and CTLA-4 (ipilimumab) (Morganstein et al., 2017). Meanwhile, the general thyroid dysfunction rate was 29% after ipilimumab treatment, 18% after PD-1 treatment, and 50% after receiving the combination (Morganstein et al., 2017). It is worth mentioning that a considerable number of patients who develop hypothyroidism have a temporary symptom of hyperthyroid at the initial phase, which highlights the importance of timely recognition and careful nursing to avoid medical negligence. Unfortunately, the pathogenesis of developing ICI-related thyroiditis still remains unknown. According to a previous study conducted by Muir et al. (2021), female individuals, younger patients, and those who undergo combination of PD-1 and CTLA-4 inhibitors have higher possibilities of developing thyroiditis. For HCC patients undergoing ICI treatments, screening for thyroid-stimulating hormone and thyroxine regularly is necessary, which allows doctors to diagnose thyroid dysfunction when the patient is still asymptomatic. In general, hypothyroidism related to ICI does not lead to the termination of treatment, and an incremental thyroid replacement therapy at a dose of 25–50 μg is adequate for treating symptomatic hypothyroidism (Sangro et al., 2013). For patients developing hyperthyroidism after ICI treatment, consultation with an endocrinologist is recommended and the heart rate should be maintained below 90 bpm (Sangro et al., 2013).

## Summary

The establishment of immunotherapy has reshaped the treatment paradigm for advanced HCC in the past decades, and more immune checkpoints, as well as the combination therapies, are being studied further. Although related to a wide range of irAEs, immunotherapy remains the key point of future research, with the hope of overcoming cancer.

## Abbreviation

HCC, hepatocellular carcinoma; HBV, hepatitis B virus; HCV, hepatitis C virus; NAFLD, non-alcoholic fatty liver disease; OS, overall survival; TKI, tyrosine kinase inhibitor; PD-1, programmed cell death-1; PD-L1, programmed cell death ligand 1; CTLA-4, cytotoxic T-lymphocyte-associated protein 4; ICI, immune checkpoint inhibitor; NSCLC, non-small-cell lung cancer; FDA, Food and Drug Administration; RECIST, Response Evaluation Criteria in Solid Tumors; ORR, objective response rate; irAEs, immune-related adverse events; PFS, progression-free survival; BSC, best supportive care; TTP, time-to-time progression.
